# A CRISPR-Cas and Tat Peptide with Fluorescent RNA Aptamer System for Signal Amplification in RNA Imaging

**DOI:** 10.3390/bios13020293

**Published:** 2023-02-18

**Authors:** Heng Tang, Junran Peng, Xin Jiang, Shuang Peng, Fang Wang, Xiaocheng Weng, Xiang Zhou

**Affiliations:** 1 Department of Clinical Laboratory, Center for Gene Diagnosis, Program of Clinical Laboratory Medicine, Zhongnan Hospital of Wuhan University, Key Laboratory of Biomedical Polymers of Ministry of Education, The Institute for Advanced Studies, College of Chemistry and Molecular Sciences, Wuhan University, Wuhan 430072, China; 2 Hubei Province Key Laboratory of Allergy and Immunology, The Institute for Advanced Studies, College of Chemistry and Molecular Sciences, Wuhan University, Wuhan 430072, China; 3 School of Pharmaceutical Sciences, Wuhan University, Wuhan 430071, China

**Keywords:** CRISPR, Tat peptide, RNA aptamer, RNA imaging

## Abstract

We reported on an efficient RNA imaging strategy based on a CRISPR-Cas and Tat peptide with a fluorescent RNA aptamer (TRAP-tag). Using modified CRISPR-Cas RNA hairpin binding proteins fused with a Tat peptide array that recruits modified RNA aptamers, this simple and sensitive strategy is capable of visualizing endogenous RNA in cells with high precision and efficiency. In addition, the modular design of the CRISPR-TRAP-tag facilitates the substitution of sgRNAs, RNA hairpin binding proteins, and aptamers in order to optimize imaging quality and live cell affinity. With CRISPR-TRAP-tag, exogenous *GCN4*, endogenous mRNA *MUC4*, and lncRNA *SatIII* were distinctly visualized in single live cells.

## 1. Introduction

RNA-seq technologies have been mushrooming over the years, and they have helped researchers to understand the significant role of RNA even further [1]. While tools for RNA profiling were developing, RNA visualizing approaches in living cells also shed light on the study of the functions and dynamics of RNA. Recently, the CRISPR systems, with excellent RNA targeting mechanisms demonstrated by applications in the genome or in transcriptome editing, have been wildly applied for intracellular RNA imaging [2,3,4,5,6,7]. Fluorescent proteins (FPs) can be fused to nuclease-dead mutations of Cas9 or Cas13 [8,9] to localize RNAs of interest with the help of a guide RNA and light them up via efficient FP signals. The strategy of the CRISPR–FP conjugate has shown its superiority for RNA visualization and the tracing of RNA dynamics in live cells, but there still remains plenty of potential to upgrade [6]. PAMer sequences need to be inserted in target RNAs for Cas9 recognition, which may influence the original status of the RNA. Apart from that, the archaea-derived Cas proteins have a large size and high immunogenicity, which may interfere with their efficiency and durability in live cells. More recently, CRISPR-Cas-inspired RNA targeting system (CIRTS)-based approaches have been explored [10,11], applying guide RNAs and RNA binding proteins of human origin, but it is not clear as to whether the general engineering of programmable RNA effector proteins is suitable for RNA imaging.

There is more room for the improvement of CRISPR–FP, wherein the unbound fluorescent proteins induce an unavoidable fluorescence background signal, which makes this strategy require multiplex fusions of FP to obtain amplified signals. To optimize, the RNA aptamers exhibiting bright and stable fluorescence, when combined with the fluorophore-like synthetic dye [12], could be a potential candidate for replacing FPs to produce a fluorescent signal. They are easily engineerable, and they produce relatively low backgrounds because of the lower stability of uncombined RNA double-stranded structures in live cells [7,13,14,15]. Recently, many fluorescent RNA aptamers have been developed [16,17,18,19]. Unfortunately, the artificial insertion of a number of hairpins into the target RNA may interfere with the distribution and behaviors of endogenous RNAs, similar to the MS2-MCP system [20,21,22]. Modifying CRISPR-guided RNA with aptamer insertion has been demonstrated to visualize both DNA and RNA [13,15]; yet, the signal amplification is mainly based on increasing the quantity of aptamer structures, which is limited by whether this sgRNA-aptamer could be correctly expressed and folded. Thus, it is vital to introduce a new dimension to amplify the signal in the strategy that combines the RNA aptamer and the CRISPR system.

Multiple peptides in series interacting with other proteins via an antigen–antibody connection, such as the Suntag system, have been fused into the Cas protein to recruit function proteins for site-specific transcriptome modifications, such as methylation, for DNA as well as for RNA [23,24]. These multiple peptides provide a wide operation window and an improved degree of efficiency that is attributed to the aggregation of function proteins, which inspires us to explore their potential for signal amplification in imaging. There also exist peptides with specific structures that can compactly interact with distinct RNA secondary structures, represented by an arginine-rich RNA-binding peptide, Tat. It could interact with the trans-activation response (TAR) hairpin variation 2, providing an accurate assembly of the peptides–RNA complex [25,26].

**Scheme 1:**
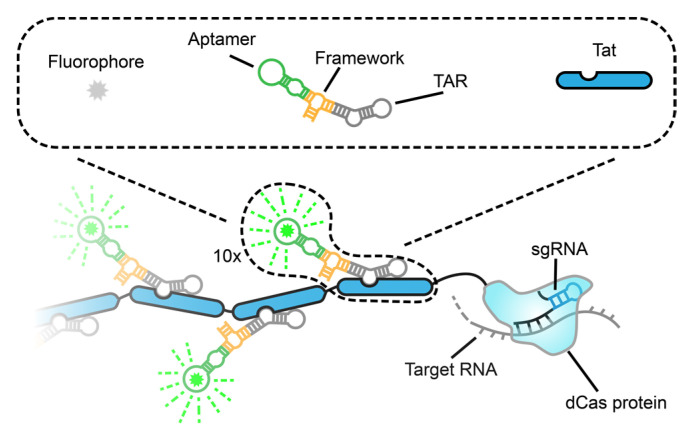
Development of a strategy for RNA imaging via the combination of Tat peptide with fluorescent RNA aptamer (TRAP-tag). dCas13 protein is tagged with Tat peptides series, which is capable of recruiting RNA aptamer attached to TAR (trans-activation response).

Hence, we suppose that engineering a TAR-aptamer that combines a peptide series will be feasible for introducing a dimension to amplify the signal while possessing the advantage of the aptamer. Here, in this work, we designed a strategy for RNA imaging via the combination of the Tat peptide with a fluorescent RNA aptamer named TRAP-tag (Scheme 1). It may integrate accurate RNA recognition using the dCas13 system and the potentially high imaging efficiency produced by fluorescent aptamers aggregating on Tat arrays. The signal amplification is achieved not only by using an aptamer series but also by increasing the quantity of Tat in another dimension. TAR–aptamer structures are expected to be extremely unstable, and they are easily degraded via cellular processes, which may wipe out a part of the background fluorescence produced by the dissociated TAR–aptamer conjugate in the cellular environment. Moreover, the system is easily programmable due to its flexibility, which means that the dCas13 protein could be replaced by smaller and less invasive RNA binding proteins in CIRTS, reducing the immunogenicity of the imaging system. The modifiable TAR–aptamer design also provides alternative choices of different types of aptamers, which may be attributed to further applications in multicolor RNA imaging.

## 2. Materials and Methods

### 2.1. Design of the CRISPR-10 × Tat and TAR–Aptamer

To design a stable RNA scaffold that is compatible with the integration of multiple RNA aptamers and TAR, we used the consensus sequence F30 [27]. The detailed designs of TAR-Broccoli, TAR-2 × Broccoli, TAR-Pepper, and TAR-8 × Pepper are shown in Table S1. MFold [28] was applied to simulate the folding of the designed RNA aptamer sequence and to calculate the suboptimal free energy (SFE) and the minimum free energy.

### 2.2. Plasmid Construction

pmRuby3-HSF1, pmRuby3-24 × GCN4, and pHAGE-dPspCas13b-3 × EGFP-2 × NLS-IRES-puro were from Ling-Ling Chen. pHAGE-dPspCas13b-10 × Tat-2 × NLS-IRES-puro were constructed from pHAGE-dPspCas13b-3 × EGFP-2 × NLS-IRES-puro by removing 3 × EGFP and replacing it with 10 × Tat. Pm1374-CIRTS 3 and Pm1374-CIRTS 10 were synthesized by GenScript. pHAGE-CIRTS 3-10 × Tat-2 × NLS-IRES-puro, pHAGE-CIRTS 3-3 × EGFP-2 × NLS-IRES-puro, pHAGE-CIRTS 10-3 × EGFP-2 × NLS-IRES-puro, and pHAGE-CIRTS 10-10 × Tat-2 × NLS-IRES-puro were constructed from pHAGE-dPspCas13b-3 × EGFP-2 × NLS-IRES-puro and pHAGE-dPspCas13b-10 × Tat-2 × NLS-IRES-puro.

A series of TAR-Aptamer cassettes were cloned into the pLKO.5 plasmid after the U6 promoter between the BmgBI and EcoRI restriction enzyme cutting sites. Then, we performed Golden Gate Assembly to insert specific spacer sequences into two BbsI sites behind the U6 promoter and before the transcription start sites of single guide RNA cassettes, as shown in detail in Figure S1. All repetitive spacers of the target sites and sgRNA hairpin sequences are listed in Table S2. The Cas-Tat and RNA cassettes’ expression vectors reported here will be deposited at Addgene.

### 2.3. Cell Cultivation and Transfection

Human embryonic kidney cell line 293T (Dingguo changsheng Biotechnology Co., Ltd., Beijing, China) were cultured in 20 mm glass-bottom dishes (Nest Corporation, London, UK) with high glucose DMEM (Gibco, Waltham, MA, USA), in addition to 10% (*v/v*) FBS at 37 °C and 5% CO_2_. For transfection, we used Lipofectamine 3000 (Thermo Fisher Scientific, Waltham, MA, USA) to co-transfect 200 ng each of dCas-10 × Tat-2 × NLS and 1 μg each of plasmid DNA for the desired guide RNAs and TAR-aptamer. Additionally, before imaging, the cells were put into another 24–48 h of incubation.

### 2.4. Single-Molecule Fluorescence In Situ Hybridization (smFISH)

The Stellaris Probe Designer was used to design all of the smFISH probes labeled with Cy5 on the 3′ ends (Table S3). An RNA FISH experiment was performed, as previously reported [29]. In short, 4% PFA was firstly used to fix cells for 15 min, and then the cells were washed three times by DPBS, followed by permeabilization with 70% EtOH overnight and three rounds of washing with DPBS. Cells were incubated in 10% formamide/2 × SSC for 15 min at 50 °C, and after that, hybridization was achieved at 30 °C for 16 h.

### 2.5. Confocal Laser Scanning Microscopy

Cells cultured in confocal vessels and transfected with desired plasmids were imaged after the removal of the medium and staining with a fluorophore. Images were acquired with a microscope (DMIRB; Leica Biosystems) that possesses an EMC CD camera (iXon-897D; Andor Technology) equipped with a 63 × oil objective lens (NA 1.4) and a 2× magnification adapter. Basically, 405, 488, 552, and 638 nm excitation lights were used for the DAPI, Broccoli/Pepper, mRuby3, and smFISH fluorescence excitations, respectively. Correspondingly, 415–478 nm, 498–542 nm, 562–618 nm, and 650–700 nm emission signals were acquired. All of the fluorescence imaging data of the cells were analyzed using Fiji/ImageJ [30] (https://imagej.net/Welcome, accessed on 18 June 2020). The signal-to-noise ratio (SNR) was calculated according to Figure S2.

### 2.6. Fluorophore Synthesis

DFHBI-1T was purchased from Sigma, and HBC530 was synthesized following the protocols given in a previous report [20].

HBC530, ^1^H NMR (400 MHz, DMSO-*d6*) δ 8.01 (s, 1 H), 7.93 (s, 2 H), 7.90 (s, 2 H), 7.86 (d, *J* = 8.7 Hz, 2 H), 6.85 (d, *J* = 9.1 Hz, 2 H), 4.77 (t, *J* = 5.3 Hz, 1 H), 3.59 (t, *J* = 5.3 Hz, 2 H), 3.53 (t, *J* = 5.6 Hz, 2 H), 3.07 (s, 3 H). ^13^C NMR (101 MHz, DMSO-*d6*) δ 152.09, 145.93, 140.15, 133.39, 132.43, 125.90, 120.51, 119.37, 119.22, 111.96, 110.08, 100.17, 58.67, 54.27, 39.23.

## 3. Results and Discussion

### 3.1. Verification of the RNA Imaging Feasibility of TRAP-Tag in Live Cells

**Figure 1:**
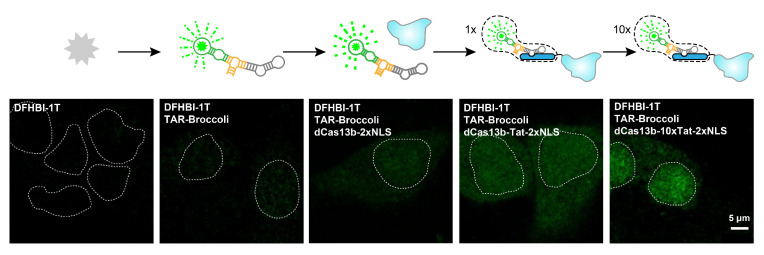
Verification of the feasibility of the CRISPR-TRAP-tag (CRISPR-Cas and Tat peptide with a fluorescent RNA aptamer) in live cells. Top, schematic of the CRISPR-TRAP-tag system to amplify the signal. Bottom, images of CRISPR-TRAP-tag in the 293T cells. First column, negative control. Second column, pTAR-Broccoli were transfected to the cells. Third column, the cells were co-transfected with pdPspCas13b-2 × NLS and pTAR-Broccoli. Fourth column, the cells were co-transfected with pdPspCas13b-Tat-2 × NLS and pTAR-Broccoli. Fifth column, the cells were co-transfected with pdPspCas13b-10 × Tat-2 × NLS and pTAR-Broccoli. The cell was treated with DFHBI-1T (10 μM) before imaging. Scale bar, 5 μm. The nucleus was dyed by DAPI (not shown), and the outline was illustrated as a dashed line.

Broccoli, a well-known fluorescent RNA aptamer that can combine and enable the (Z)-4-(3,5-difluoro-4-hydroxybenzylidene)-2-methyl-1-(2,2,2-trifluoroethyl)-1*H*-imidazol-5(4*H*)-one) DFHBI-1T to produce a green fluorescence, was selected to verify the feasibility of the strategy. In another aspect, we chose the Tat peptide, an ideal object for recruiting TAR RNA with distinct structures [25,26], to enhance the signal in imaging RNAs. Thus, firstly, the TAR scaffold was fused with Broccoli aptamers using a well-designed framework [27] (Figure 1 top, Table S1). We asked whether this TAR-Broccoli was capable of folding in a correct and stable structure for a light-on mechanism by DFHBI-1T in living cells. A detectable degree of fluorescence by employing TAR-Broccoli and DFHBI-1T simultaneously (Figure 1, first and second columns) was witnessed, which indicates the successful light-on of the Broccoli module in the scaffold. However, the fluorescence intensity was relatively low. Next, we constructed a dCas protein with a Tat, and the results showed that its fluorescence was brighter than dCas without Tat (Figure 1, third and fourth columns), which may be due to the enhanced stability of the aptamer after binding the protein. This indicates that the modified aptamer and dCas protein fused peptide could also bind well. Remarkably, when dPspCas13b fused with 10 × Tat, tag was simultaneously transfected and obvious green, fluorescent signals could be observed (Figure 1, fifth column), demonstrating that the TAR-Broccoli RNAs interacted with the Tat peptides array to form a dPspCas13b-TRAP-tag complex. Coupling aptamers with the RNA-aggregating capability of the 10 × Tat peptides array significantly enhanced the brightness and the photostability of the fluorescence signal.

### 3.2. Labeling Exogenous GCN4 mRNAs Using TRAP-Tag

**Figure 2:**
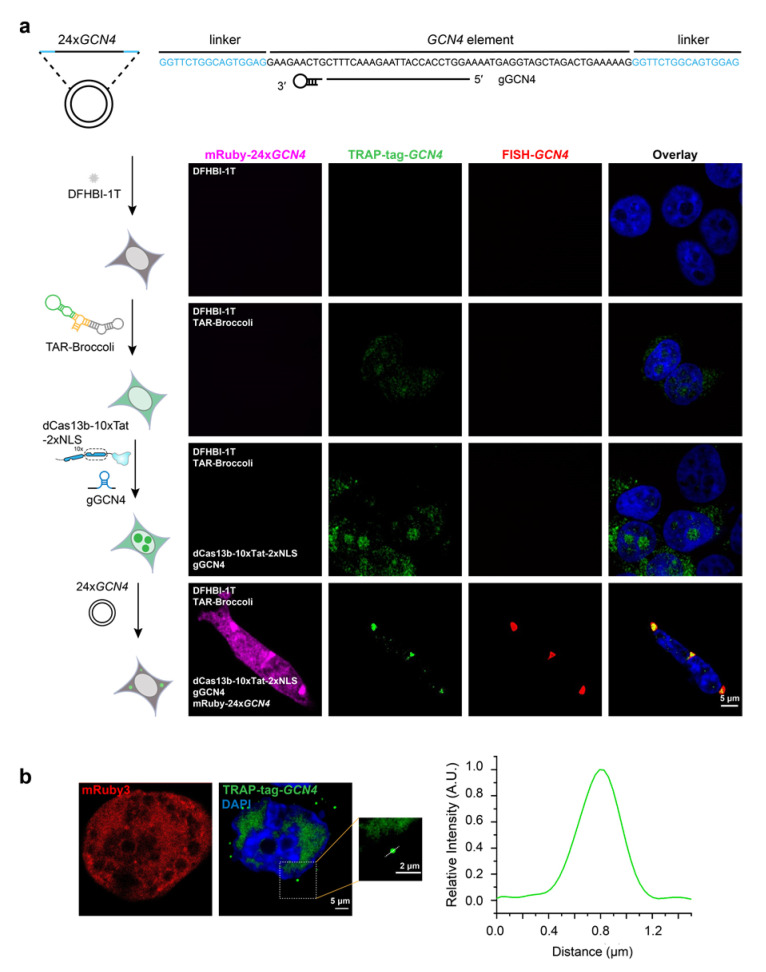
Labeling of exogenous *GCN4* mRNAs using the CRISPR-TRAP-tag. (**a**) Schematic and image of applying the CRISPR-TRAP-tag to label *GCN4* mRNAs. The sgRNA (single guide RNA) targeting repeats in *GCN4* are shown. Images of the 24 × *GCN4* labeled using TRAP-tag. First row, negative control; Second row, pTAR-Broccoli were transfected to the cells; Third row, cells were co-transfected with pdPspCas13b-10 × Tat-2 × NLS, pgGCN4, and pTAR-Broccoli. Bottom row, co-transfection of pdPspCas13b-10 × Tat-2 × NLS, pgGCN4, pTAR-Broccoli, and pmRuby3-24 × *GCN4* was carried out. The nucleus was dyed using DAPI. (**b**) Partially enlarged image of TRAP-tag labeling *GCN4* in 293T cells. The dotted line in the right panel indicates the line scan of the relative fluorescence intensity of the signal. The nucleus was dyed using DAPI. The diameter of the light point was about 0.5 μm. A scale bar is shown in the figure. The cell was treated with DFHBI-1T (10 μM).

Next, we evaluated the RNA imaging ability of the CRISPR-TRAP-tag inside the cell. To ensure that the quantity of repeats was suitable for early-stage experiments, we chose a construct containing 24 × *GCN4* elements to express RNAs with abundant repeats as the target. The 24 × *GCN4* repeats were initially designed as a protein tag for signal amplification through antibody binding [31], but they have also been used in the preliminary assessment of other RNA imaging approaches [6,13]. The dCas13b sgRNA was designed to target 24 × *GCN4* to study the feasibility and sensitivity of this system in live cells (Figure 2a). To image *GCN4* RNA localized in the nucleoplasm, a 2 × Nuclear Localization Signal (NLS) was fused to the system to bring it into the cell nucleus [6]. A step-by-step confirmation was carried out, and it indicated the successful formation of dPspCas13b-TRAP-tag in the presence of sgRNA (Figure 2a, first three rows). Meanwhile, the accuracy and efficiency of the CRISPR-TRAP-tag system visualizing *GCN4* were confirmed via colocalization with single-molecule FISH (smFISH) (Figure 2a, bottom row, Figures S2 and S3). The enlarged image (Figure 2b, left) illustrated that the dPspCas13b-TRAP-tag generated the fluorescent signals, light points with a diameter of about 0.5 μm (Figure 2b, right), which likely benefit from the smaller size of the fluorogenic dyes and the larger MW of the CRISPR-TRAP-tag, which makes them less diffusible. For this experiment, 2 × NLS was absent, so the signal occurred mainly in the cytoplasm (Figure 2b), demonstrating that this system is capable of imaging RNA in different intracellular locations.

### 3.3. Tracking Endogenous MUC4 mRNAs and Long Non-Coding RNA SatIII Using TRAP-Tag

**Figure 3:**
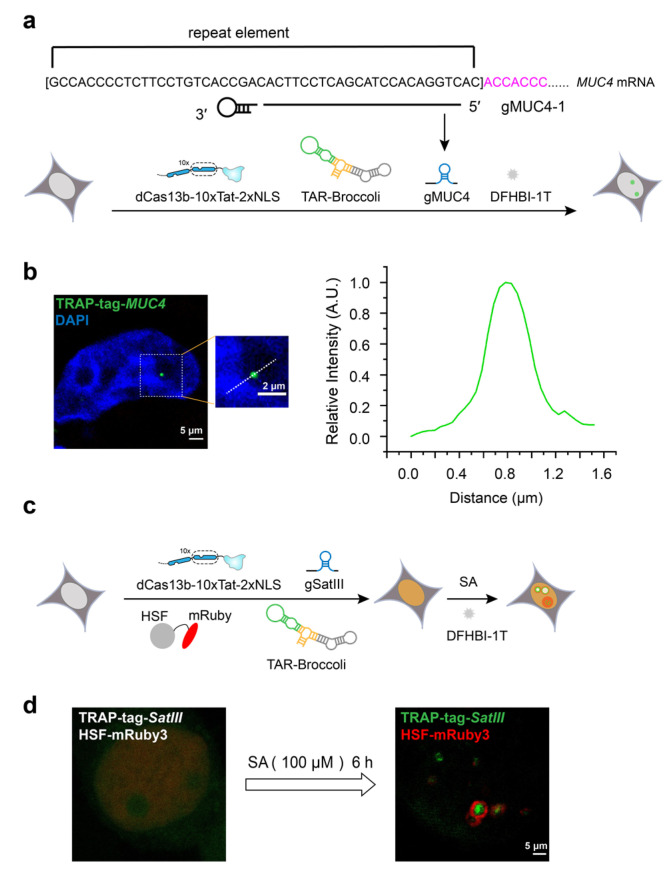
Tracking endogenous *MUC4* mRNAs and long non-coding RNA *SatIII* via TRAP-tag. (**a**) Schematic of applying CRISPR-TRAP-tag to label *MUC4* mRNAs. (**b**) Left, images of 293T cells co-transfected with pdPspCas13b-10 × Tat-2 × NLS, pgMUC4, and pTAR-Broccoli. The nucleus was dyed using DAPI and treated with DFHBI-1T (10 μM); Right, relative fluorescence intensity of signal was scanned as the dotted line in the right panel. The diameter of the light point is about 0.5 μm. Scale bar is shown in the figure. (**c**) Schematic of applying CRISPR-TRAP-tag to tracking *SatIII* lncRNA under SA treatment. (**d**) Images of the same cell before (left) and after (right) SA treatment; the cell is co-transfected with pHSF-mRuby3, pdPspCas13b-10 × Tat-2 × NLS, pgSatIII, and pTAR-Broccoli, and is treated with DFHBI-1T (10 μM).

Membrane mucin MUC4 is a demonstrated diagnostic marker in cancer that expresses in many epithelial cells, and it plays a protective role [32]. Thus, visualizing the distribution and localization of MUC4 transcripts are essential for understanding MUC4 expression and function. The successful GCN4 imaging encouraged us to test the ability of our approach to visualize endogenous mRNAs. We designed gRNA that can base-pair with the repeat’s region in exon 2 of MUC4 mRNA [33,34] (Figure 3a). Not surprisingly, detectable signals could be observed (Figure 3b and Figure S4). Meanwhile, the colocalization result showed a 0.5 μm diameter signal, indicating the accuracy of the CRISPR-TRAP-tag, even if the target RNA is endogenous in low abundance. When eukaryotic cells are exposed to stressful conditions, a large number of RNA–protein assemblies will emerge. Under thermal or chemical stresses—for example, sodium arsenite (SA) treatments—the heat shock transcription factor 1 (HSF1)-dependent activation of Satellite III (*SatIII*) transcription occurred, and long non-coding RNA Satellite III (*SatIII*) was gathered to produce nuclear stress bodies (nSBs) [35]. An RNA imaging method may help to reveal the composition and dynamics of ribonucleoprotein (RNP) granules to further interpret their mechanisms. We built up gRNA based on previous studies targeting SatIII lncRNA to confirm whether our system was suitable for visualizing RNA in nSBs (Figure 3c). When cells were treated using SA, we obtained signals that were more aggregated than in the untreated condition. This typical aggregation of SatIII surrounded by the red HSF1-mRuby3 fluorescence signal (Figure 3d) is consistent with previous reports [6,35]*,* which indicated the association between the loci of *SatIII* and HSF to form nuclear stress bodies. By contrast, no gathered signal of TAR-Broccoli was witnessed in the absence of sgRNA (Figure S5). These results showed that CRISPR-TRAP-tag is applicable for investigating lncRNA and for tracking the formation of RNP granules.

### 3.4. TRAP-Tag Can Combine with other CRISPR Proteins in RNA Imaging

**Figure 4:**
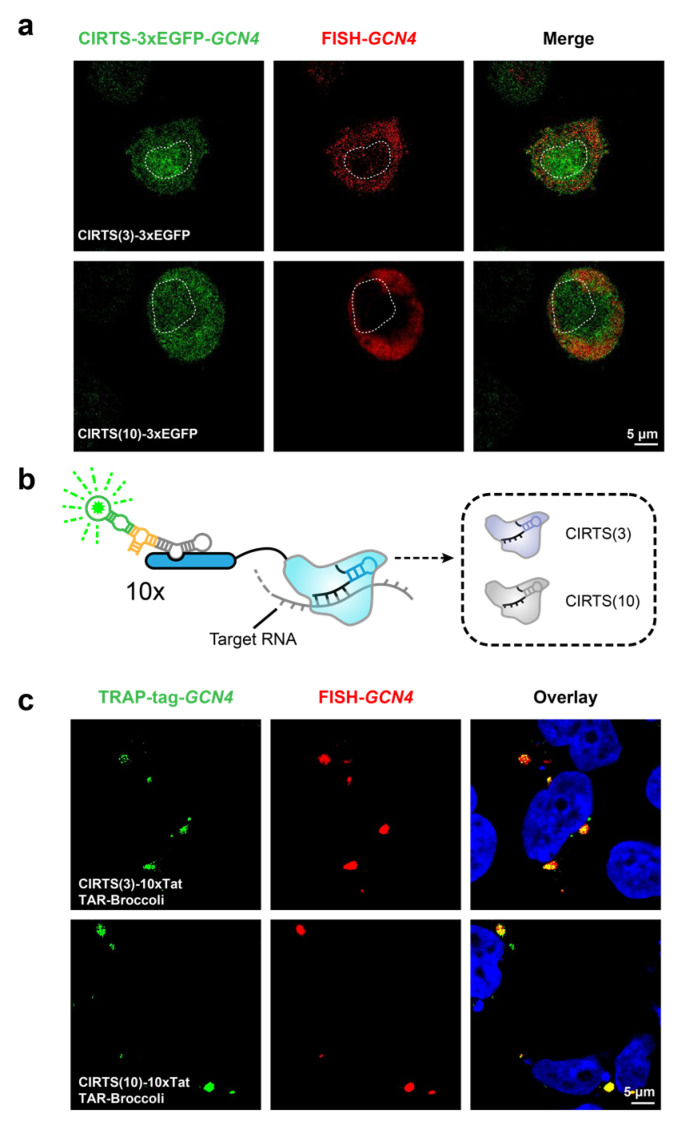
Combination of CIRTS(3)/CIRTS(10) with TRAP-tag for *GCN4* RNA imaging. (**a**) Representative images of *GCN4* labeled using CIRTS(3)-3 × EGFP and CIRTS(10)-3 × EGFP with gRNAs. (**b**) Schematic of combination CIRTS(3)/CIRTS(10) with TRAP-tag. (**c**) Representative images of *GCN4*-labeled using CIRTS(3)/CIRTS(10) and TRAP-tag with gRNAs.

Although the Cas13 systems revolutionarily promoted the research on RNA, the huge size and archaea source of these proteins become major obstacles in their applications of both fundamental research and clinical practice. To date, most Cas13 proteins are around 130 kDa. Recently, a strategy named the CRISPR-Cas-inspired RNA targeting system (CIRTS) has been developed to surmount the large size and immunogenicity of present RNA targeting strategies by applying protein parts from the human proteome [10]. Similar to the CRISPR-Cas system, CIRTS is composed of a ribonucleoprotein and a gRNA, which interacts with the target transcriptome via Watson-Crick-Franklin base-pairing, delivering the whole protein complex site-specifically to the RNA [10]. However, when directly fusing 3 × EGFP to both the human hairpin-binding protein U1A (TBP6.7) in CIRTS(3) and the human histone stem-loop binding protein (SLBP) in CIRTS(10), the imaging capability was somehow unsatisfying to visualize exogenous GCN4 when sgRNAs corresponding to the RNA hairpin binding proteins were also transfected (Figure 4a). This result could be due to the high backgrounds of 3 × EGFP and the misfolding of the fused proteins. We then replaced 3 × EGFP with our TRAP-tag, connecting them to CIRTS(3) and CIRTS(10) to image GCN4 (Figure 4b). Similarly to the dPspCas13b-TRAP-tag, the CIRTS(3) and CIRTS(10)-TRAP-tag successfully colocalized with the smFISH signals of GCN4 (Figure 4c), demonstrating that the TRAP-tag is suitable for the CIRTS system in order to reduce the size and immunogenicity, while the accuracy and SNR of imaging are guaranteed.

### 3.5. CRISPR-TRAP-Tag Systems Adaptive to another RNA Aptamer

**Figure 5:**
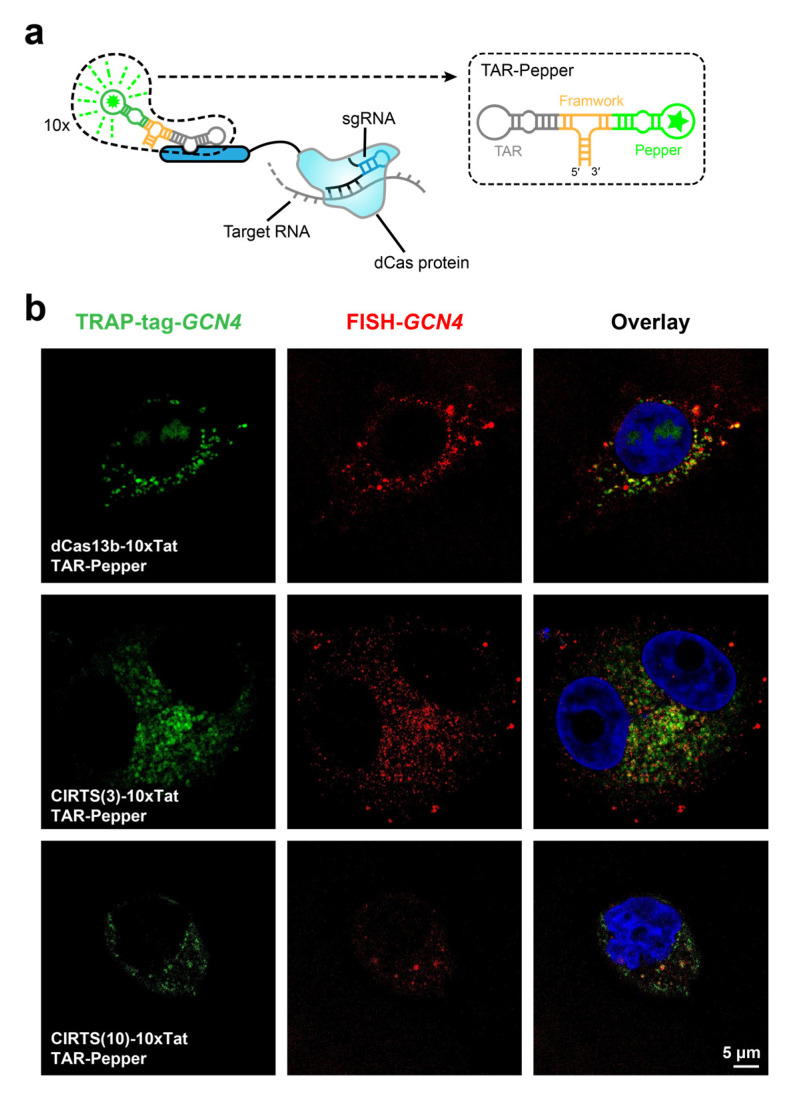
CRISPR-TRAP-tag systems were compatible with the RNA aptamer Pepper. (**a**) Scheme of the strategy for Pepper version of CRISPR-TRAP-tag systems. (**b**) Representative images of GCN4 labeled using CRISPR-TRAP-tag with RNA aptamer Pepper with gRNAs.

Represented by the CIRTS replacement of dPspCas13b, as indicated above, the flexibility of the modularized design is one of the advantages of the CRISPR-TRAP-tag. The fluorescence RNA aptamer part of the TAR-aptamer RNA could also be easily substituted using other species of aptamer. We replaced the aptamer component of the CRISPR-TRAP-tag with another species of RNA aptamer named Pepper, attributed to its smaller size and its higher and steadier fluorescence signal, with a red-to-cyan range of emission wavelength [20] (Figure 5a). HBC530, a GFP fluorophore-like synthetic dye, can interact with TAR-Pepper’s aptamer part to produce green fluorescence. Exogenous GCN4 can be successfully imaged using the CRISPR-TRAP-tag system with TAR-Pepper, no matter whether the RNA hairpin binding part is dPspCas13b, CIRTS(3), or CIRTS(10), with distinct fluorescent signals (Figure 5b). Furthermore, the colocalization of smFISH with the imaging verified the targeting accuracy, suggesting the capability of precise RNA imaging.

### 3.6. CRISPR-TRAP-Tag Systems with Multiplex RNA Aptamers Improve the Signal-To-Noise Ratio (SNR)

**Figure 6:**
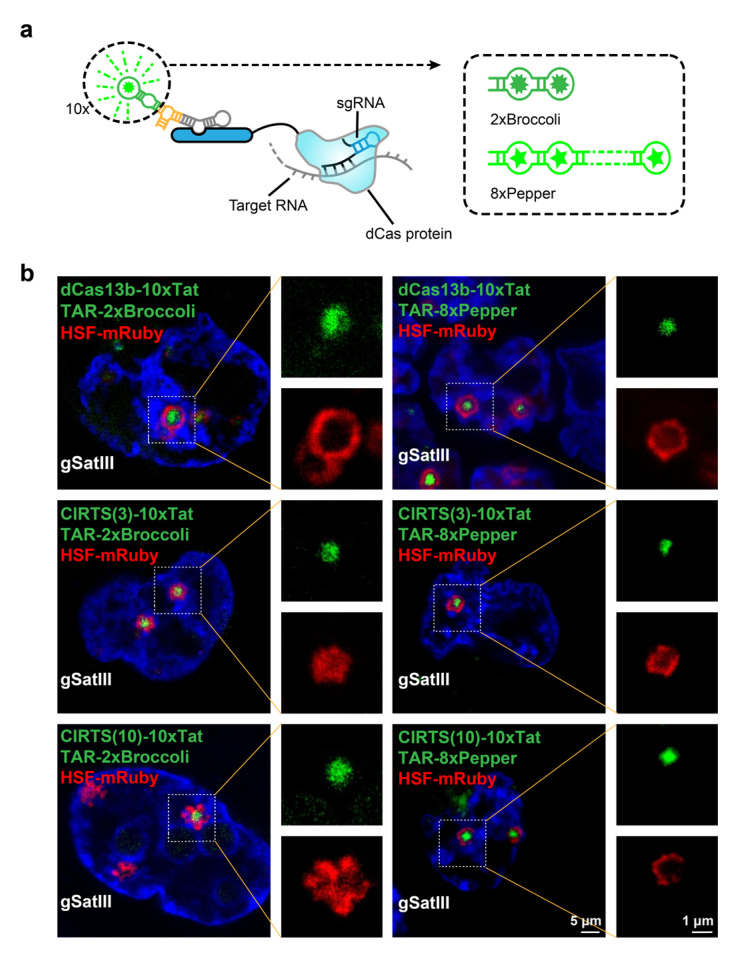
CRISPR-TRAP-tag systems with multiplex RNA aptamers. (**a**) Diagram of the strategy for CRISPR-TRAP-tag systems with multiplex RNA aptamers. (**b**) Representative images of *SatIII* labeled using CRISPR-TRAP-tag, with multiplex RNA aptamers with gRNAs.

Lastly, benefitting from the advantages of RNA aptamers that have a small size, it may be feasible to introduce a multiplex aptamers array to visualize a target with a brighter fluorescence and a higher SNR. Therefore, we doubled the Broccoli and octupled the Pepper aptamers using connecting frameworks and then connected both of them to the TAR scaffold (Figure 6a, Table S1). TAR connecting multiple RNA aptamers could be aggregated using multiple Tat peptides, theoretically resulting in an increased signal strength attributed to both the 2 ×/8 × aptamer and the 10 × Tat array. The CRISPR-TRAP-tag system with 2 × Broccoli and 8 × Pepper targeting *SatIII* lncRNA resulted in a similar relative localization with HSF-mRuby as indicated before, which verified the imaging capability of the CRISPR-TRAP-tag with the multiplex RNA aptamer (Figure 6b). Furthermore, according to the SNR analysis of *SatIII* targeting (Figure S6), all six systems with multiple aptamers applications resulted in excellent SNRs. Notably, the systems possessing CIRTS(3) and CIRTS(10) had a relatively higher SNR compared to that possessing dPspCas13b, indicating that the CIRTS overcomes the large size and bacterial-derived defects of dCas13b to provide higher performance in our imaging strategy. On the other hand, systems with 8 × Pepper produced a higher SNR than those with 2 × Broccoli.

## 4. Conclusions

In summary, CRISPR-TRAP-tag was demonstrated as an ideal RNA localizing and visualizing method in living cells, considering the aspects of its conciseness, dynamic range, sensitivity, and flexibility. The continuous increase of the types of fluorescent light-up aptamers provides more flexibility for the CRISPR-TRAP-tag system for simultaneously imaging multiple RNAs with different colors. This strategy provides a new method to investigate RNA distribution and has several aspects of merit. Firstly, the CRISPR-TRAP-tag system is applicable for RNA imaging, such as cytoplasmic mRNA and intranuclear lncRNA in live cells, providing a multipurpose platform (Figure 2 and Figure 3). Secondly, we, for the first time, found that the CIRTS(3) and CIRTS(10) fused with 3 × EGFP were unable to track exogenous *GCN4* RNA with high abundance, which might be due to the high backgrounds of 3 × EGFP and probable protein misfolding. However, CRISPR-TRAP-tag-fused TBP/SLBP exhibited a smaller and more accurate fluorescent signal (Figure 4). Thus, the proposed strategy is not only suitable for the dPspCas13b protein, but it also offers a general RNA imaging methodology in live cells for CIRTS. Thirdly, this strategy has a modular design that can be extended by simply replacing the modules, such as protein, aptamer, or peptide (Figure 4 and Figure 5). The multiplex design of the RNA aptamer leads to a higher fluorescence brightness and SNR, improving the sensitivity of RNA imaging (Figure 6). Due to these features, CRISPR-TRAP-tag possesses feasibility and applicability to RNA imaging that can further enhance our understanding of cellular RNA dynamics and functions.

## Data Availability

Data are contained within the article.

## Supplementary Materials

The following supporting information can be downloaded at: https://www.mdpi.com/article/10.3390/bios13020293/s1. Table S1. Sequences of TAR-Aptamer. Sequences are showed in the corresponding color of modules in the figures below (TAR in gray, framework in orange, Broccoli in green and Pepper in neon green). Multiple aptamer sequences are connected with 3-basepair-linkers and units of them are indicated as superscript serial-number and underline. DFHBI-1T and HBC530 fluorophores are revealed as green stars. Table S2. Sequences of sgRNA, targeting *GCN4*, *MUC4* and *SatIII*. Sequences are showed in the corresponding color of modules in the figures below (spacer sequences in black, dCas13 sgRNA hairpin in blue, TBP sgRNA hairpin in purple and SLBP sgRNA hairpin in gray). Table S3. *GCN4* smFISH probes (5′-3′). Figure S1. Single-step cloning for sgRNA cassettes in Table S2 with guide sequence to label RNA target. Guide sequence oligo (black) was inserted after cutting of BbsI restriction enzyme to gener-ate a seamless cloning of guide sequence in the upstream of sgRNA hairpin sequence (blue). Figure S2. Schematic for the calculation of SNR. Figure S3. Colocalization analysis of TRAP-tag-GCN4 and smFISH signals in HEK 293T cells. The TRAP-tag composed by dCas13b-10 × Tat-2 × NLS, sgRNA targeting GCN4 and TAR-Broccoli are expressed in the cells to visualize GCN4. Left, image of the GCN4 foci. Right, line scan of the relative fluorescence intensity of the signal indicated as the dotted line in the image on the left. Figure S4. Labeling endogenous MUC4 mRNAs by the CRISPR TRAP-tag, including control groups of Figure 3b. First row, dPspCas13b-10 × Tat-2 × NLS were transfected to the cells; Second row, the cells were co-transfected with pdPspCas13b-10 × Tat-2 × NLS and pTAR-Broccoli. Bottom row, co-transfection of pdPspCas13b-10 ×Tat-2 × NLS, pgMUC4 and pTAR-Broccoli was carried out. The nucleus was dyed by DAPI. Scale bar is shown in the figure. The cell was treated with DFHBI-1T (10 μM) before imaging. Figure S5. Representative images of HSF1-mRuby3 (red) and TAR-Broccoli (green) in HEK 293T cells upon SA (100 μM, 6 h) treatment. Top row, cell expressing HSF-mRuby3 is transfected with TAR-Broccoli and treated by DFHBI-1T(10 μM) to image after SA treatment, as a blank control of dCas protein for the experiment in Figure 3d. Bottom row, dPspCas13b-10 × Tat-2 × NLS and TAR-Broccoli (without sgRNA of dCas13b targeting SatIII) were transfected to the cells expressing HSF-mRuby3, followed by SA and DFHBI-1T treatments, as a blank control of sgRNA for the ex-periment in Figure 3d. Figure S6. Signal-to-Noise Ratio (SNR) in Figure 6. The labels below the x-axis refer to the components of TRAP-tag, indicating the dCas protein and apta.
