# Supplementary material for: A CRISPR-Cas and Tat Peptide with Fluorescent RNA Aptamer System for Signal Amplification in RNA Imaging
Source: Biosensors (Basel). 2023 Feb 18;13(2):293. doi: 10.3390/bios13020293 (PMC9954185; doi:10.3390/bios13020293)
Supplement: Supplementary file 1 [file biosensors-13-00293-s001.zip › biosensors-2173312-supplementary.pdf]

biosensors

MDPI

Supplementary Material

A CRISPR-Cas and Tat peptide with Fluorescent RNA Aptamer System for Signal Amplification in RNA Imaging

Heng Tang [1,†], Junran Peng [1,†], Xin Jiang [1], Shuang Peng [1], Fang Wang [2], Xiaocheng Weng [1,*] and Xiang Zhou [1]

Table S1. Sequences of TAR-Aptamer. Sequences are showed in the corresponding color of modules in the figures below (TAR in gray, framework in orange, Broccoli in green and Pepper in neon green). Multiple aptamer sequences are connected with 3-basepair-linkers and units of them are indicated as superscript serial-number and underline. DFHBI-1T and HBC530 fluorophores are revealed as green stars.

	**TAR-Aptamer Sequences (5′-3′)**
TAR-Broccoli	UUGCCAUGUGUAUGUGGGGGCUCGUGUAGCUCAU-<br>UAGCUCCGAGCCCCCACAUACUCUGAU-<br>GAUCCGAGACGGUCGGGUCCAGAUAUUCGUAUCUGUCGAGUAGAGUGU<br>GGGCUCGGAUCAUUCAUGGCAA
TAR-2xBroccoli	UUGCCAUGUGUAUGUGGGGGCUCGUGUAGCUCAU-<br>UAGCUCCGAGCCCCCACAUACUCUGAU-<br>GAUCC[1]GAGACGGUCGGGUCCAUCU[2]GAGACGGUCGGGUCCAGAUAUUC<br>GUAUCUGUCGAGUAGAGUGUGGGCUCAGA[1]UGUCGAGUAGAGUGUG-<br>GGCUCGGAUCAUUCAUGGCAA
TAR-Pepper	UUGCCAUGUGUAUGUGGGGGCUCGUGUAGCUCAU-<br>UAGCUCCGAGCCCCCACAUACUCUGAU-<br>GAUCCCCAAUCGUGGCGUGUCGGCCUGCUUCGGCAGGCACUGGCGCC<br>GGGAUCAUUCAUGGCAA
TAR-8xPepper	UUGCCAUGUGUAUGUGGGGGCUCGUGUAGCUCAU-<br>UAGCUCCGAGCCCCCACAUACUCUGAU-<br>GAUCC[1]CCAAUCGUGGCGUGUCGGCCUCUC[2]CCAAUCGUGGCGUGUCGG<br>CCUCUC[3]CCAAUCGUGGCGUGUCGGCCUCUC[4]CCAAUCGUGGCGUG-<br>UCGGCCUCUC[5]CCAAUCGUGGCGUGUCGGCCUCUC[6]CCAAUCGUG-<br>GCGUGUCGGCCUCUC[7]CCAAUCGUGGCGUGUCGGCCU-<br>CUC[8]CCAAUCGUGGCGUGUCGGCCUCUCUUCGGA-<br>GAGGCACUGGCGCCGGAG[7]AGGCACUGGCGCCGGAG[6]AG-<br>GCACUGGCGCCGGAG[5]AGGCACUGGCGCCGGAG[4]AGGCACUGGCGCCG-<br>GAG[3]AGGCACUGGCGCCGGAG[2]AGGCACUGGCGCCGGAG[1]AG-<br>GCACUGGCGCCGGGAUCAUUCAUGGCAA

Modular structures of TAR-Aptamers (TAR in gray, framework in orange, Broccoli in green and Pepper in neon green, DFHBI-1T and HBC530 fluorophores are revealed as green stars).

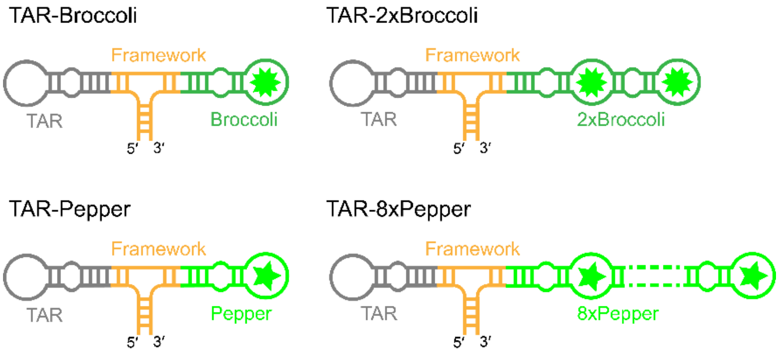

Table S2. Sequences of sgRNA, targeting *GCN4*, *MUC4* and *SatIII*. Sequences are showed in the corresponding color of modules in the figures below (spacer sequences in black, dCas13 sgRNA hairpin in blue, TBP sgRNA hairpin in purple and SLBP sgRNA hairpin in gray).

		sgRNA sequences (5′-3′)
gGCN4	dCas13b	GGTGGTAATTCTTTGAAAGCAGGUUGUG-GAAGGUCCAGUUUUGAGGGGCUAUUACAAC
	CIRTS(3)	GGTGGTAATTCTTTGAAAGCAGGGCCAGATCTGAGCCTGG-GAGCTCTCTGGCC
	CIRTS(10)	GGTGGTAATTCTTTGAAAGCAGCCAAAGGCTCTTCTCAGAGCCACCCA
gMUC4	dCas13b	GTGACCTGTGGATGCTGAGGGUUGUG-GAAGGUCCAGUUUUGAGGGGCUAUUACAAC
	CIRTS(3)	GTGACCTGTGGATGCTGAGGGGCCAGATCTGAGCCTGG-GAGCTCTCTGGCC
	CIRTS(10)	GTGACCTGTGGATGCTGAGGCCAAAGGCTCTTCTCAGAGCCACCCA
gSatIII	dCas13b	GATTCCAATCCATGCCATTCCACGUUGUG-GAAGGUCCAGUUUUGAGGGGCUAUUACAAC
	CIRTS(3)	GATTCCAATCCATGCCATTCCACGGCCAGATCTGAGCCTGG-GAGCTCTCTGGCC
	CIRTS(10)	GATTCCAATCCATGCCATTCCACCCAAAGGCTCTTCTCAGAGCCACCCA

Modular structures of sgRNA (spacer sequences in black, dCas13 sgRNA hairpin in blue, TBP sgRNA hairpin in purple and SLBP sgRNA hairpin in gray).

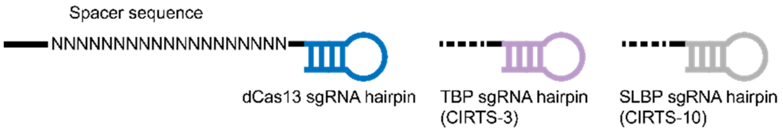

Table S3. *GCN4* smFISH probes (5′-3′).

	***GCN4* smFISH probe (5′-3′)**
GCN4-sm-1	GCAGTTCTTCTCCACTGCCAGAA
GCN4-sm-2	GGTGGTAATTCTTTGAAAGCAG
GCN4-sm-3	GCTACCTCATTTTCCAGGTGG

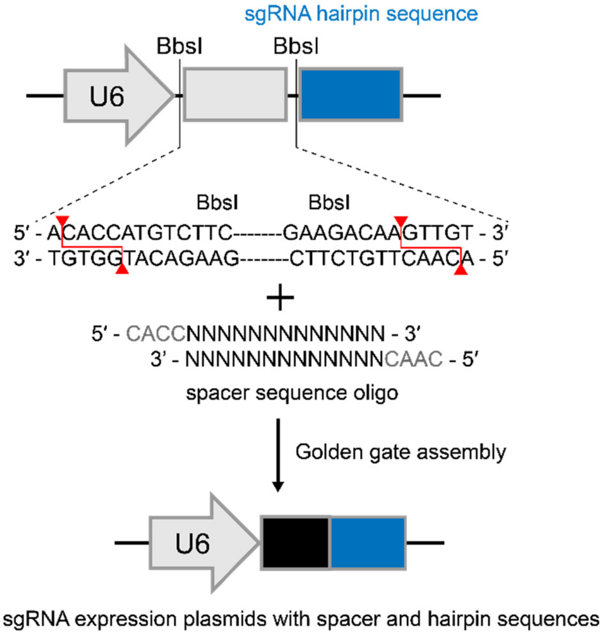

Figure S1. Single-step cloning for sgRNA cassettes in Table S2 with guide sequence to label RNA target. Guide sequence oligo (black) was inserted after cutting of BbsI restriction enzyme to generate a seamless cloning of guide sequence in the upstream of sgRNA hairpin sequence (blue).

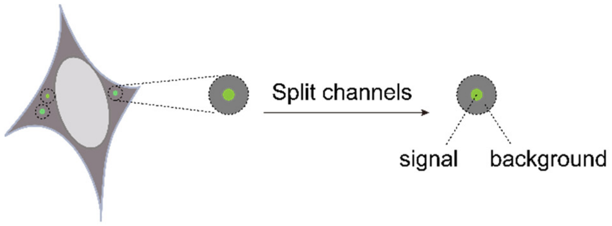

$$\text{Signal to noise ratio} = P_{signal} / P_{noise} = \frac{\text{Max intensity of signal - Mean intensity of background}}{\text{Std. dev. of background signal}}$$

Figure S2. Schematic for the calculation of SNR.

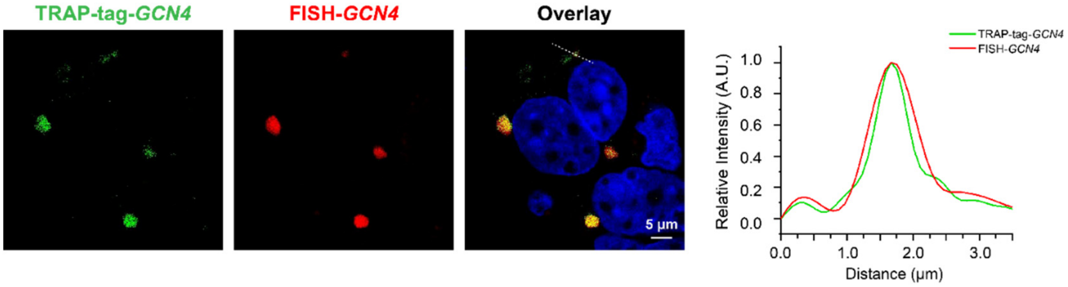

Figure S3. Colocalization analysis of TRAP-tag-*GCN4* and smFISH signals in HEK 293T cells. The TRAP-tag composed by dCas13b-10xTat-2xNLS, sgRNA targeting GCN4 and TAR-Broccoli are expressed in the cells to visualize GCN4. Left, image of the *GCN4* foci. Right, line scan of the relative fluorescence intensity of the signal indicated as the dotted line in the image on the left.

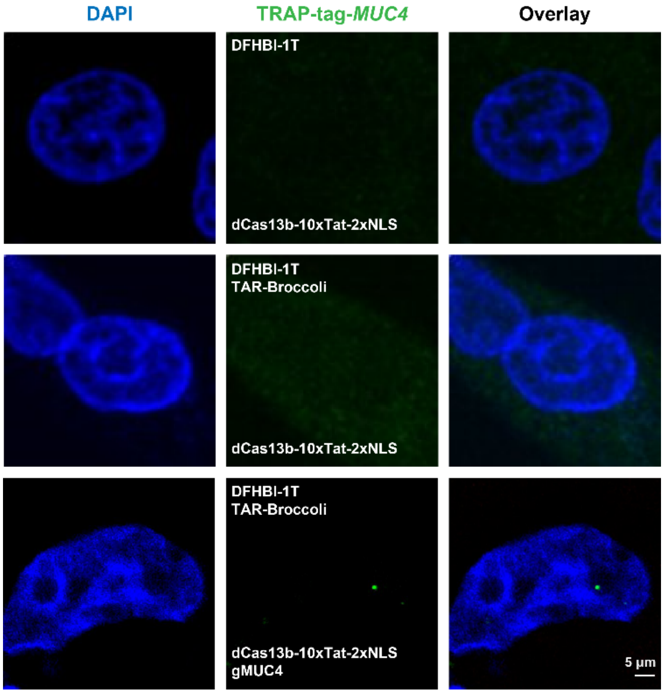

Figure S4. Labeling endogenous *MUC4* mRNAs by the CRISPR TRAP-tag, including control groups of Figure 3b. First row, dPspCas13b-10xTat-2xNLS were transfected to the cells; Second row, the cells were co-transfected with pdPspCas13b-10xTat-2xNLS and pTAR-Broccoli. Bottom row, co-transfection of pdPspCas13b-10xTat-2xNLS, pgMUC4 and pTAR-Broccoli was carried out. The nucleus was dyed by DAPI. Scale bar is shown in the figure. The cell was treated with DFHBI-1T (10 μM) before imaging.

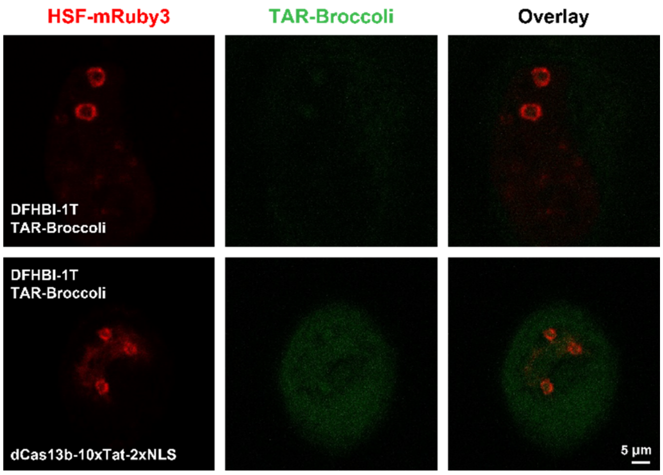

Figure S5. Representative images of HSF1-mRuby3 (red) and TAR-Broccoli (green) in HEK 293T cells upon SA (100 μM, 6 h) treatment. Top row, cell expressing HSF-mRuby3 is transfected with TAR-Broccoli and treated by DFHBI-1T(10uM) to image after SA treatment, as a blank control of dCas protein for the experiment in Figure 3d. Bottom row, dPspCas13b-10xTat-2xNLS and TAR-Broccoli (without sgRNA of dCas13b targeting *SatIII*) were transfected to the cells expressing HSF-mRuby3, followed by SA and DFHBI-1T treatments, as a blank control of sgRNA for the experiment in Figure 3d.

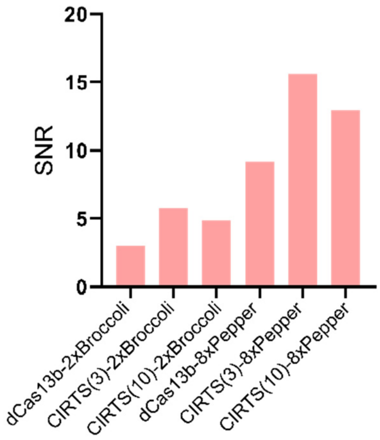

Figure S6. Signal-to-Noise Ratio (SNR) in Figure 6. The labels below the x-axis refer to the components of TRAP-tag, indicating the dCas protein and apta.
